# Determinants of first and second trimester induced abortion - results from a cross-sectional study taken place 7 years after abortion law revisions in Ethiopia

**DOI:** 10.1186/s12884-014-0416-9

**Published:** 2014-12-19

**Authors:** Kristine Ivalu Bonnen, Dereje Negussie Tuijje, Vibeke Rasch

**Affiliations:** Department of Obstetrics and Gynaecology, Odense University Hospital, Odense, Denmark; Department of Obstetrics and Gynaecology, Jimma University Specialized Hospital, Jimma, Ethiopia

**Keywords:** First trimester abortion, Second trimester abortion, Unsafe abortion, Contraception, Ethiopia

## Abstract

**Background:**

In 2005 Ethiopia took the important step to protect women’s reproductive health by liberalizing the abortion law. As a result women were given access to safe pregnancy termination in first and second trimester. This study aims to describe socio-economic characteristics and contraceptive experience among women seeking abortion in Jimma, Ethiopia and to describe determinants of second trimester abortion.

**Methods:**

A cross-sectional study conducted October 2011 - April 2012 in Jimma Town, Ethiopia among women having safely induced abortion and women having unsafely induced abortion. In all 808 safe abortion cases and 21 unsafe abortion cases were included in the study. Of the 829 abortions, 729 were first trimester and 100 were second trimester abortions. Bivariate and multivariate logistic regressions were used to determine risk factors associated with second trimester abortion. The associations are presented as odds ratios (OR) with 95% confidential intervals. Age stratified analyses of contraceptive experience among women with first and second trimester abortions are also presented.

**Results:**

Socio-economic characteristics associated with increased ORs of second trimester abortion were: age < 19 years, being single, widowed or divorced, attending school, being unemployment, being nullipara or para 3+, and having low education. The contraceptive prevalence rate varied across age groups and was particularly low among young girls and young women experiencing second trimester abortion where only 15% and 19% stated they had ever used contraception.

**Conclusion:**

Young age, poor education and the prospect of single parenthood were associated with second trimester abortion. Young girls and young women were using contraception comparatively less often than older women. To ensure women full right to control their fertility in the setting studied, modern contraception should be made available, accessible and affordable for all women, regardless of age.

## Background

In 2008, an estimated 92% of African women of childbearing age lived in countries with restrictive abortion laws [1]. Since 1997, eight African countries have changed their abortion laws reducing the restrictions (Benin, Chad, Ethiopia, Guinea, Mali, Niger, Swaziland and Togo) [2]. The most liberal abortion laws in Africa are found in Cape Verde and South Africa where pregnancy termination is allowed without restriction as to reason and Zambia which permits abortion on socio-economic grounds. In South Africa the abortion law was liberalized in 1997 and a 91% reduction in the annual number of abortion-related deaths were observed between 1994 and 2000 [3,4]. However, seventeen years after South Africa adopted one of the world’s most progressive abortion laws, women’s access to safe services is becoming more difficult and unsafe abortion appears to be on the rise [5]. In 2005, Ethiopia took the important step to protect women’s reproductive health by liberalizing the abortion law from 1955, which only permitted abortion to save the pregnant woman from grave and permanent danger to life. Termination of pregnancy is now also permitted where continuation of the pregnancy or birth endangers the health or life of the woman or the child, in cases of fetal impairment, if the woman has physical or mental disabilities or is a minor who is not physically or psychologically prepared to raise a child, or where pregnancy is a result of rape or incest. Under these circumstances abortion can legally be induced up to 28 weeks of gestational age.

Unwanted pregnancy is a present problem in Ethiopia as illustrated in a national quantitative study of abortion incidence where it was estimated that out of 4 million pregnancies, 42% were unwanted leading to 382,000 induced abortions [6]. The newly implemented abortion law enables women, who experience an unwanted pregnancy, to have a safe first or second trimester abortion. Studies have described the availability and quality of safe abortion service in Ethiopia after the abortion law reform. The findings show that in some areas safe abortion care service fell far short of the recommended levels [7] whereas other areas have experienced an increase in availability of safe abortion care service [8] and a corresponding decrease in treatment of incomplete abortion [9].

Abortion history and contraceptive use after the abortion law reform have been described in studies focusing on female students. According to these studies high abortion rates and low contraceptive prevalence rates prevail [10-12]. However, female students are a highly selected group of young individuals and to get a more nuanced picture of the abortion situation in Ethiopia there seems to be a need of a more general description of women who are seeking abortion. Further, since second trimester abortion may be associated with greater distress and higher rates of morbidity and mortality [13], attention should be given to this particular group of women. Against this background the present study aims to describe socio-economic characteristics and contraceptive use among women seeking first and second trimester abortion in Jimma Town and describe risk factors associated with second trimester abortion. Such information may help inform the Ethiopian family planning programme to attune their service to better reach women at risk of abortion and more specifically women at risk of second trimester abortion.

## Methods

### Study setting

Jimma Town has a population of 120,960 people and is the capital town of Jimma Zone which has a population of 2,486,155 people [14]. In 2011, a total of 4634 first trimester and 195 second trimester abortions were according to available registers performed in Jimma Town.

The present study was conducted in four health facilities in Jimma Town: Jimma Health Centre, Marie Stopes International Ethiopia, Family Guidance Association of Ethiopia and Jimma University Specialized Hospital.

### Study population

During the period Oct 2011-April 2012 women having first and second trimester abortion and women admitted with complications after an unsafe abortion were invited to participate in the study.

**Figure 1:**
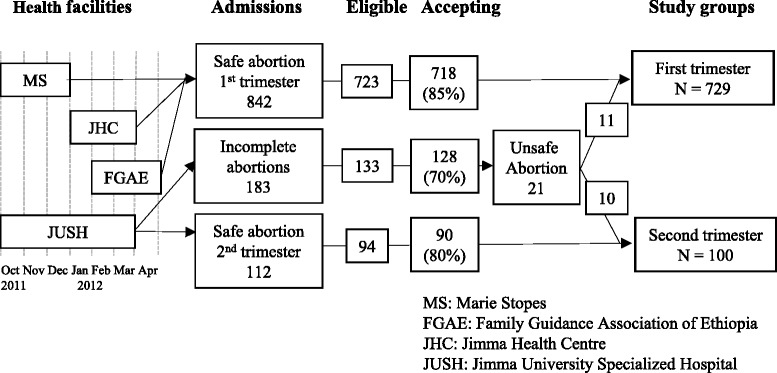
**Recruitment of the study population.**

First trimester abortion: Women were recruited from three health facilities in Jimma town that offered safe abortion in first trimester (Jimma Health Centre, Marie Stopes International Ethiopia, Family Guidance Association of Ethiopia). Data were collected for 3 months at each health facility. Out of 842 admitted women, 723 were eligible for interview and 718 (85% of admitted) accepted to participate in the study (Figure 1).

Second trimester abortion: Women were recruited at Jimma University Specialized Hospital, the only health facility in Jimma town which offered second trimester abortion. Out of 112 admitted women, 94 were eligible for interview, and 90 (80% of admitted) accepted to participate in the study.

Unsafe abortion: Women admitted with incomplete abortion at Jimma University Specialized Hospital underwent an empathic interview to determine if the incomplete abortion was a result of an unsafely induced abortion. The interviews were conducted as a confidential in-depth dialogue without any questionnaire or notes. When the interviewer felt she had gotten trustworthy information as to whether the woman had undergone an unsafe induced abortion, the informant was asked permission to record the obtained information in a structured questionnaire. We have previously with success used this approach to identify women with unsafe abortion in Tanzania [15-17]. In all 183 women were admitted with incomplete abortion, 133 of these women were eligible for interview, the remaining had been discharged before they could be approached. Out of 133 eligible women, 128 (70% of the admitted) accepted participation. In all 21 (16%) women stated they were admitted due to complications after either a legal abortion (n = 11) or an illegal abortion (n = 10) (Figure 1).

In sum, 808 women with safely induced abortions were included in the study together with 21 women who had experienced an unsafely induced abortion. The small number of unsafe abortions did not allow independent analysis. Of the 829 abortion cases, 729 were performed in first trimester and 100 in second trimesters (Figure 1).

### Variables

A structured questionnaire was used to obtain information on socio-economic characteristics and contraceptive use. Socio-economic variables relating to induced abortion included age (<19 years, 20–24 years, 25+ years) marital status (single, married, widowed/divorced), education level (primary school or less, secondary school or above), occupation (housewife, employed, unemployed, attending school, other occupation), place of residence (Jimma Town, Jimma Zone, other) and parity (nullipara, para I-II, para III+). To explore whether women having induced abortion had previous experience using contraception and to what extend the unintended pregnancy was a result of contraceptive failure the following variables were included; ever use of contraception (condom or modern contraception such as contraceptive pill, injection, implant or IUD), and use of contraception in relation to the intercourse where conception most likely happened.

### Statistical analysis

Data were double entered in EpiData Entry 3.1 and exported to the statistical software package Stata Release 12.1 (Stata Corp. College Station, TX) for statistical analysis. The profiles of women having abortion are presented as frequencies. First and second trimester induced abortion were compared using first trimester abortions as the reference group. The associations are presented as odds ratio (OR) with 95% confidence intervals (CI). Socio-economic variables were controlled for confounding by logistic regression analysis, including risk factors from the bivariate analysis with P-values of < 0.05 (age, marital status, education, place of residence, parity). Since education and occupation are closely related variable, only educational level was included in the logistic regression analysis. The multivariate analysis was repeated for women with residence in Jimma Town in order to control for the effect of place of residence.

### Ethics

The women were explained the purpose of the study and were assured confidentiality. Written consent was obtained from all women accepting participation in the study. Ethical clearance was obtained from Jimma University Ethical Review Committee of the College of Public Health and Medical Science.

## Results

### Socio-economical profile, women having first and second trimester abortion

The median age was 21 years for women having first trimester abortion and 18 years for those having second trimester abortion (Table 1). Women aged 20–24 years represented 46% of women obtaining abortion in first trimester. Adolescents (<19 years) represented 56% of those having abortion in second trimester. Single women accounted for 48% of the first trimester abortions and 80% of the second trimester abortions. Women who had education at secondary school level or above represented 68% of women having abortion in first trimester, whereas they only represented 32% of those having abortion in second trimester. Girls and young women attending school accounted for 42% of the first trimester abortions and 57% of the second trimester abortions. Jimma Town was the place of residence for 64% of women having first trimester abortion and for 41% of those experiencing second trimester abortion. Women who came from outside Jimma Zone accounted for 4% of women having first trimester abortion and 17% of women having second trimester abortion. Women who had not previously given birth represented 66% of those experiencing a first trimester abortion and 84% of the women experiencing a second trimester abortion.

**Table 1:** **Socio-economic characteristics of women with first and second trimester induced abortion**

	**First trimester abortion**	**Second trimester abortion**	**Second trimester vs first trimester abortion**	**Second trimester vs first trimester abortion**
	**N = 729%**	**N = 100%**	**OR (95% CI)**	Adjusted* OR (95% CI)
**Age**				
Median (rang)	21 (14–42)	18 (14–35)		
<19 years	191/729 26.2	55/99 55.6	**3.77 (1.92-7.39)**	**2.64 (1.23-5.68)**
20-24 years	334/729 45.8	27/99 27.3	1.06 (0.52-2.14)	1.20 (0.57-2.55)
25+ years	204/729 28.0	17/99 17.2	1.00	1.00
**Marital status**				
Single	345/726 47.5	80/100 80.0	**6.90 (3.62-13.1)**	**6.55 (3.02-14.2)**
Married	357/726 49.2	12/100 2.0	1.00	1.00
Widowed/Divorced	24/726 3.3	8/100 8.0	**9.92 (3.56-27.6)**	**7.31 (2.50-21.4)**
**Education**				
< sec school	233/727 32.0	67/99 67.7	**4.44 (2.78-7.19)**	**6.07 (3.66-10.1)**
Sec school+	494/727 68.0	32/99 32.3	1.0	1.00
**Occupation**				
Housewife	145/727 19.9	1/99 1.01	**0.10 (0.002-0.71)** ǂ	**0.07 (0.01- 0.56)**
Employed	197/727 27.1	13/99 13.1	1.00	1.00
Unemployed	10/727 1.4	9/99 9.1	**13.6 (4.31-43.2)**	**10.1 (2.92-35.0)**
Attending school	305/727 42.0	56/99 56.6	**2.78 (1.47-5.26)**	**2.35 (1.10-5.00)**
Other	70/727 9.6	20/99 20.2	**4.33 (1.99- 9.37)**	1.85 (0.79-4.32)
**Place of residence**				
Jimma Town	463/727 63.7	41/100 41.0	1.00	1.00
Jimma Zone	235/727 32.3	42/100 42.0	**2.02 (1.27-3.20)**	**1.71 (1.04-2.81)**
Other	9/727 4.0	17/100 17.0	**6.62 (3.28-13.4)**	**9.13 (4.20-19.8**)
**Parity**				
0	481/729 66.3	84/100 84.0	**4.30 (2.03-9.13)**	**2.55 (1.17-5.56)**
1-2	197/729 27.1	8/100 8.0	1.00	1.00
3+	48/729 6.6	8/100 8.0	**4.10 (1.44-11.7)**	**7.09 (2.41 -20.9)**

Significant results are marked in Bold.

ǂFisher’s exact (all other: chi2).

*Adjusted for; Age, Marital status, Education, Place of residence, Parity.

Missing values are not shown in the columns, and therefore column sum do not add up with total in top row.

### Risk factors associated with second trimester abortion

Multivariate analysis was used to ascertain the independent effect of each socio-economic variable on the outcome, second trimester abortion, after controlling for confounders. The multivariate analysis showed that the OR of having abortion in second trimester relative to having abortion in first trimester, were two times greater for women aged 19 years or younger compared to women aged 25+ years (OR = 2.64% CI: 1.23-5.68) (Table 1). The OR of having the abortion in second trimester was six times higher for single women in comparison with married women (OR = 6.55% CI: 3.02-14.2). Similarly, widowed or divorced women had seven times increased OR of having abortion in second trimester relative to having abortion in first trimester (OR = 7.31; 95% CI: 2.50-21.4). The odds of having a second trimester abortion were six times greater for women having education on primary school level or below compared to women who had education at secondary school level or above (OR: 6.07; 95% CI: 3.66-10.1). Occupational situation was associated with second trimester abortion. Women or girls who were still attending school had in comparison with employed women, a twice increased OR of having second trimester abortion (OR = 2.35; 95% CI: 1.10-5.00). To be a housewife seemed to be a protective factor for having second trimester abortion, (OR = 0.07; 95% CI: 0.01-0.56). Unemployment was found to be associated with a ten time increased OR for second trimester abortion (OR = 10.1; 95% CI: 2.92-35.0). For women with residence in Jimma Zone the odds of having second trimester abortion relative to having first trimester abortion, were nearly twice that of women with residence in Jimma Town (OR = 1.7; 95% CI: 1.04-2.81). The odds of having second trimester abortion were nine times greater for women with residence outside Jimma Zone compared with women with residence in Jimma Town (OR = 9.13, 95% CI; 4.20-19.8). Finally, a significant association between parity and second trimester abortion was found. Women who were nullipara had in comparison to women who were para I-II, a more than twice increased OR of having second trimester abortion relative to having abortion in first trimester (OR = 2.55; 95% CI 1.17-5.56). Women who were para III or more had a seven times greater OR of having second trimester abortion (OR: 7.09; 95% CI: 2.41-20.9).

The multivariate analysis was repeated for women with residence in Jimma Town only, and showed that for this group marital status and education level were associated with second trimester abortion. Among women with residence in Jimma Town the OR of having second trimester abortion relatively to having first trimester abortion were five times greater for single women compared with married women (OR: 5.07; 95% CI: 1.71-15.0) (Table 2). For the same group the OR of having second trimester abortion were four times greater for women having education on primary school level or below compared to women having education at secondary school level or above (OR: 4.08; 95% CI: 2.00-8.33).

**Table 2:** **Socio-economic characteristics of women with residence in Jimma Town having first and second trimester induced abortion**

	**First trimester abortion**	**Second trimester abortion**	**Second trimester vs first trimester abortion**	**Second trimester vs first trimester abortion**
	**N = 463%**	**N = 41%**	**OR (95% CI)**	Adjusted* OR (95% CI)
**Age**				
Median (rang)	21 (14–38)	18 (14–27)		
<19 years	119/463 25.7	25/41 61.0	**6.93 (2.27-21.2)**	2.88 (0.82-10.2)
20-24 years	212/463 45.8	12/41 29.3	1.87 (0.59-5.93)	1.30 (0.36- 4.62)
25+ years	132/463 28.5	4/41 9.8	1.00	1.00
**Marital status**				
Single	221/461 48.0	35/41 85.4	**7.13 (2.68-18.9)**	**5.07 (1.71-15.0)**
Married	225/461 48.8	5/41 12.2	1.00	1.00
Widowed/Divorced	15/461 3.5	1/41 2.4	3.00 (0.33-27.59)	2.42 (0.23-25.0)
**Education**				
< sec school	139/462 30.1	24/41 58.5	**3.28 (1.63-6.71)**	**4.08 (2.00-8.33)**
Sec school+	323/462 69.9	17/41 41.5	1.00	1.00
**Occupation**				
Housewife	80/463 17.3	1/41 2.4	0.20 (0.004- 1.56)ǂ	0.28 (0.03- 2.58)
Employed	129/463 27.9	8/41 19.5	1.00	1.00
Unemployed	5/463 1.1	3/41 7.3	**9.68 (1.82-51.3)**	4.02 (0.61-26.3)
Attending school	201/463 43.4	25/41 61.0	**2.00 (1.87-4.60)**	1.22 (0.45-3.30)
Other	48/463 10.4	4/41 9.8	1.34 (0.38-4.69)	0.50 (0.13-1.96)
**Parity**				
0	309/463 67.0	37/41 90.3	**5.07 (1.56-26.1)**ǂ	2.16 (0.53-8.78)
1-2	127/463 27.6	3/41 7.3	1.00	1.00
3+	25/463 5.4	1/41 2.4	**1.69 (0.31-22.0)**ǂ	2.99 (0.27-33.6)

Significant results are marked in Bold.

ǂFisher’s exact (all other: chi2).

*Adjusted for; Age, Marital status, Education, Parity.

Missing values are not shown in the columns, and therefore column sum do not add up with total in top row.

### Contraception

**Figure 2:**
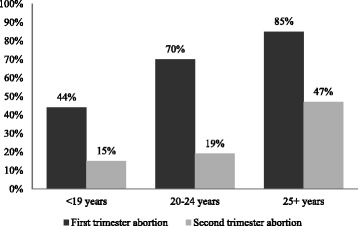
**Percentage of women experienced using contraception by age group.**

**Figure 3:**
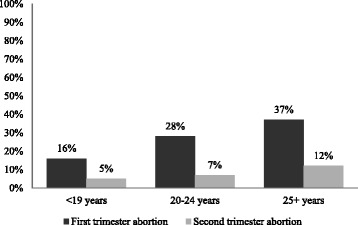
**Percentage of women using contraception in relation to intercourse where conceived by age group.**

Ever use of modern contraception was reported by 67% of the women having first trimester induced abortion and 21% of the women having second trimester induced abortion (Table 3). Ever use of contraception by age groups is presented in Figure 2. The frequency in contraceptive use increased by age among both women having first and second trimester abortion, however, across all age groups the proportion of women who stated they had ever used contraception was comparatively lower among second trimester abortion women. In the age group < 19 years, 44% of the girls having first trimester abortion had ever used contraception whereas the same applied for only 15% of the girls having second trimester abortion. Among young women aged 20–24, 70% of first trimester and 19% of second trimester abortion women had ever used contraception. Among women aged 25+, 85% of first trimester and 47% of second trimester abortion women had ever used contraception. In relation to the intercourse where conception most likely happened a similar picture was found (Figure 3). In the age group < 19 years, 16% of the girls having first trimester abortion and 5% of the girls having second trimester abortion stated they had used contraception in relation to the intercourse where they conceived. Among young women aged 20–24, the same applied for 28% having first trimester and 7% having second trimester abortion whereas 37% and 12% of women aged 25+ with first trimester and second trimester abortion stated the same. Hence, the proportion of women, who stated contraceptive use at the intercourse where they most likely conceived, increased by age among both groups of abortion women but across all age groups the prevalence rates were significantly lower among women having second trimester abortion.

**Table 3:** **Contraception among women with first and second trimester abortion**

	**First trimester abortion**	**Second trimester abortion**
	**N = 729%**	**N = 100%**
**Ever used contraceptives**		
Yes	487/724 67.3	21/99 21.2
No	237/724 32.7	78/99 78.8
**Ever used condom**		
Yes	161/716 22.5	6/99 6.1
No	555/716 77.5	93/99 93.9
**Ever used hormonal contraceptives**		
Yes	406/728 55.8	19/100 19.0
No	322/728 44.2	81/100 81.0
**Contraceptives when conceived**		
Yes	199/725 27.5	7/100 7.0
No	526/725 72.6	93/100 93.0

Missing values are not shown in the columns, and therefore column sum do not add up with total in top row.

## Discussion

This study describes risk factors for second trimester abortion in a setting where safe abortion service has recently been made available by law. Low level of education and the prospect of single parenthood were strong predictors for second trimester abortion. The contraceptive prevalence rate varied across age groups and was particularly low among young girls and young women experiencing second trimester abortion.

Young girls aged 19 or below were overrepresented in this study where they comprised 26% of first trimester abortion and 56% of second trimester abortion women, respectively. In comparison, a study covering the period from 1990 to 2008 estimated the proportion of abortions among 15–19 year olds in Africa to be 22%, a figure which makes Africa the world region with the highest abortion rate among young girls [18]. When focusing on the age group 20–24 years, 46% among women having first trimester abortion and 27% among women having second trimester abortion belonged to this age group. In the aforementioned study, women aged 20–24 comprised 29% of all abortion in Africa. Hence, young girls and young women account for a disproportionate share of abortions in our study, a notably finding which likely reflects that young unmarried Ethiopian women, due to fear of the stigma attached to non-marital sexual activity, are having particular difficulties in avoiding unintended pregnancies. This fear may inhibit them from obtaining contraceptive services and from using methods correctly and consistently [18,19].

Single women were overrepresented among both first and second trimester abortion cases and in the comparative analysis the prospect of single parenthood was strongly associated with second trimester abortion. A tendency of delay in women’s age at marriage has been suggested to result in increased sexual activity among unmarried women, raising their risk of unintended pregnancy [6,20,21]. Single women are susceptible to induced abortion because of the stigmatizing and economic conditions that makes single motherhood highly disadvantaged. The statistics showing an almost universal childlessness among unmarried women in Ethiopia suggest the absence of any place in the society for unmarried women with children [20].

Women who had attended secondary school accounted for two-third of women having first trimester abortion whereas only one-third among women having second trimester had attended secondary school. Jimma is a university town and has a high density of students enrolled in higher education programs. This could be part of the explanation why educated women seem to be overrepresented among first trimester abortion cases. On the other hand, our findings may also reflect that educated women may be considered a group of first movers, using their rights to access safe abortion [22]. A study from North-western Ethiopia has similarly found that the number of women experiencing induced abortion increased with higher educational level [23]. Further, a recent study among Ethiopian female university and college students found that 32.1% of the students were familiar with the liberalization of the abortion law and women who were aware of the law were more likely to have experienced an induced abortion [11]. Our findings are further in line with a Nepalese study showing that in 2006, four years after legislation of the Nepalese abortion law, women’s knowledge of availability of legal abortion and providers increased with their educational level. The findings of the present study are, however, in contrast to a South African study which found no association between educational level and knowledge of the abortion legislation [24]. In the comparative analyses between women having first and second trimester abortion, women who had not attended secondary school had a six times increased risk of second trimester abortion. Hence, women having second trimester abortion did not belong to the same group of educated individuals as women having first trimester abortion did. Low educational level has previously been found to be associated with second trimester abortion in studies comparing first and second trimester abortion [25,26]. A plausible explanation for this association could be that less-educated women tend to take longer time between suspecting pregnancy and confirming it [27].

Contraceptive experience and use of contraception in relation to the intercourse where conception most likely happened were especially low among women having second trimester abortion. The women were not asked for their motivation for having an abortion and it is possible that some women having second trimester abortion as well as first trimester abortion did not use family planning at the time of conception because they wanted to become pregnant. The contraceptive experience was in particular low among young girls aged 15–19 years, where only 44% of the girls obtaining first trimester abortion and 15% of the girls obtaining second trimester abortion stated they had ever used contraception. Our findings are in contrast to a recent Ethiopian study among women receiving post abortion care, where 67% reported to have used contraceptives at least once in their life time [28]. They are also in contrast to a study conducted among secondary school students where 86% of sexually active girls reported they had ever used contraception [12]. Furthermore, our findings are in contrast to an Ethiopian study which found that 75% of undergraduate students, who had had unprotected sexual intercourse, had ever used emergency contraception [29]. Hence, the women with second trimester abortion seem to comprise a particularly group of young and poorly educated girls who are prone not to use contraception. This poor contraceptive use may reflect that many of the girls had not attended secondary school and therefore not been exposed to sexual and reproductive health education. Our findings are noteworthy and point to a grave need of contraceptive counseling and service reaching all young girls, regardless of their schooling level.

The Ethiopian abortion law is the result of several years’ effort by a coalition of health and women’s rights advocates who have worked together to ensure women better access to safe abortion. However, a liberal abortion law in a country does not guarantee access to safe abortion services. The experience from South Africa, where abortion has been legal since 1996, illustrates how implementation of a liberal abortion law may fail if there is no support from all involved stakeholders. Despite South Africa has some of the most developed government systems for the provision of abortion care, fewer than 50% of the public-sector health facilities licensed to provide abortion care are actually providing services and the number of unsafe abortion is on the rise [5]. Against this background, the present study also attempted to assess the magnitude of unsafe abortion in Jimma Town. Only 21 unsafe abortion cases were identified, equal to 16% of all admitted abortion complications. It may be questioned whether the study succeeded in covering all unsafe abortion cases. Some women may have had an unsafe abortion but not experienced any abortion complications or may have been treated at a health facility not authorised to handling abortion complications. Additionally some women may have been reluctant in revealing having had an unsafe abortion for fear of the legal consequences. On the other hand it may be argued that safe abortion is easily accessible in Jimma town, indicated by the fact that almost five thousand safely induced abortions were performed in 2011. The service is additionally being offered at reasonable price, varying form being free of charge in public health facilities and available at modest modes cost in NGO supported health facilities. It may therefore be anticipated that women in Jimma town are benefitting from the easy access to safe abortion, a factor which may prevent them from resorting to unsafe and risky interventions.

There are two noteworthy limitations of this study: representativity and the possibility of underreporting of unsafe abortions. The study was conducted in Jimma Town which is a university city and young women who are enrolled in colleges and universities are likely overrepresented in our study. The study population can therefore not be considered a representative sample of women having induced abortion in Ethiopia. However, data were collected at both public and private health facilities in Jimma Town and it achieved a high response rate. For that reason we believe that the study population can be considered representative for women obtaining safe abortion in Jimma Town. Further, to give a thorough picture of women obtaining abortion, data were also collected among women having unsafe abortion. Women who have undergone unsafe abortion are often reluctant or unwilling to discuss their experiences for fear of the consequences and may thus be underrepresented in this study. It can be argued that the determinants obtaining safe abortion are different from the determinants of obtaining unsafe abortion and the associations should thus have been analysed independently. Unfortunately, the small number of unsafe abortions did not allow for an independent analysis of unsafe abortions.

## Conclusion

Ethiopia took an important step towards addressing women’s sexual and reproductive health and rights in 2005 where the country implemented a liberal abortion law. However, to ensure women full right to control their fertility, it is essential that both safe abortion and modern contraception is available, accessible and affordable for all women. Young girls and young women accounted for a disproportional share of first and especially second trimester abortions in our study and their reported contraceptive use was strikingly low. Based on these findings, we argue that there is grave need for making contraceptive information and services available to all Ethiopian women, irrespective of age.
